# Microscopic Understanding of the Growth and Structural Evolution of Narrow Bandgap III–V Nanostructures

**DOI:** 10.3390/ma15051917

**Published:** 2022-03-04

**Authors:** Leilei Zhang, Xing Li, Shaobo Cheng, Chongxin Shan

**Affiliations:** Henan Key Laboratory of Diamond Optoelectronic Materials and Devices, Key Laboratory of Material Physics, Ministry of Education, School of Physics and Microelectronics, Zhengzhou University, Zhengzhou 450052, China; zhangleilei@gs.zzu.edu.cn (L.Z.); shaobocheng@gmail.com (S.C.)

**Keywords:** III–V nanowires, in situ technique, growth dynamics, structure–property relationship, structure evolution

## Abstract

III–V group nanomaterials with a narrow bandgap have been demonstrated to be promising building blocks in future electronic and optoelectronic devices. Thus, revealing the underlying structural evolutions under various external stimuli is quite necessary. To present a clear view about the structure–property relationship of III–V nanowires (NWs), this review mainly focuses on key procedures involved in the synthesis, fabrication, and application of III–V materials-based devices. We summarized the influence of synthesis methods on the nanostructures (NWs, nanodots and nanosheets) and presented the role of catalyst/droplet on their synthesis process through in situ techniques. To provide valuable guidance for device design, we further summarize the influence of structural parameters (phase, defects and orientation) on their electrical, optical, mechanical and electromechanical properties. Moreover, the dissolution and contact formation processes under heat, electric field and ionic water environments are further demonstrated at the atomic level for the evaluation of structural stability of III–V NWs. Finally, the promising applications of III–V materials in the energy-storage field are introduced.

## 1. Introduction

Due to the unique optical and electrical characteristics, III–V semiconductors have been recognized as the extension of Moore’s Law and have shown broad application prospect in infrared light detection [1,2], quantum computation [3], gas sensing [4], etc. [5,6]. The excellent electron transport and superior mobility presented by III–V nanomaterials [7,8] further benefit their applications in high-speed electronics. Vapor–liquid–solid (VLS) and vapor–solid–solid (VSS) mechanisms are frequently applied in the controlled synthesis of various III–V nanostructures. III–V nanomaterials generally present zinc blende (ZB), wurtzite (WZ) or mixed (ZB/WZ) phases, depending on the synthesis conditions. Due to the lack of direct insights during the growth process, the growth processes are generally investigated through post-growth characterizations, while key information related to the morphology and composition evolution of catalysts, phase selection and formation dynamics remains debatable. Thus, understanding the growth and phase transformation mechanisms during the growth process is quite essential for the controllable synthesis of III–V nanostructures. Recent investigations showed that their physical properties like electrical, mechanical, and optical properties are closely related to morphology, phase, and orientation. Therefore, revealing the structure–property relationship is quite important for the design and fabrication of high-performance devices.

**Figure 1:**
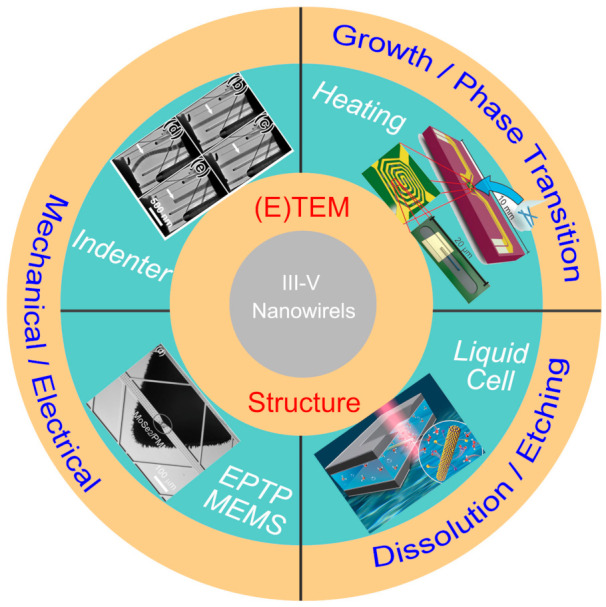
In situ microscopic techniques used in revealing the structure–property relationship of III–V nanomaterials. Top right: reproduced with permission [12]. Copyright 2016, American Chemical Society. Bottom right: reproduced with permission [13]. Copyright 2018, The Royal Society of Chemistry. Bottom left: reproduced with permission [14]. Copyright 2016, WILEY-VCH Verlag GmbH & Co. KGaA, Weinheim. Top left: reproduced with permission [15]. Copyright 2016, American Chemical Society.

In situ microscopy techniques has provided us powerful tools to reveal the underlying structural evolution under various external stimuli, [9,10] which are introduced through various holders used in the transmission electron microscope (TEM) holder and microelectromechanical system (MEMS) chips (Figure 1) [11]. In situ heating stages have been frequently used to investigate the growth mechanisms, the droplet/ nanowire (NW) interface dynamics and the metal/NW contact formation mechanisms. With a liquid cell, the dissolution and etching behavior of III–V materials under various environments can be dynamically evaluated. The mechanical and electromechanical properties of III–V materials is quite important for their practical applications, while a method of correlating these properties with their morphology and structure remains quite challenging. The development of in situ TEM technique provides solutions to realize the structure–property relationship. With the combined in situ techniques, the III–V materials have been investigated at the atomic level from synthesis to applications. 

To present a clear view of the “synthesis–structure–property” relationship, this review will start with the types, structures, and the general growth mechanisms of various III–V nanostructures. Then, the detailed structure–property relationship of III–V NWs are introduced. Firstly, we summarized the dynamic structural evolution under external fields (thermal and electrical) revealed by in situ techniques, which includes the evolution of catalysts/droplets and their roles in controlling the synthesis and phase selection processes, the contact formation mechanisms, and energy storage mechanisms. Then, the structural (orientation, phase, doping and size) dependence of various physical properties of III–V NWs are subsequently introduced. This review can provide an in-depth understanding of III–V NWs from synthesis, design and applications.

## 2. Growth of III–V Nanostructures: Synthesis and Structure

The bulk of III−V materials typically possess ZB structure [16], while III–V nanostructures generally exhibit ZB and WZ polymorphisms. The narrow bandgap materials like InAs (0.36 eV), GaAs (1.43 eV) and InSb (0.17 eV), etc. [17,18], present high carrier mobility and have shown broad applications in nanoelectronics and infrared detection [1,2,19]. III–V nanomaterials are generally synthesized through the metal-organic chemical vapor deposition (MOCVD), molecular beam epitaxy (MBE) and chemical vapor deposition (CVD) methods. For the MOCVD method, organic compounds of group-III elements and hydrides of group-V elements are used as growth sources. The gas epitaxial growth of nanostructures is realized on the substrate through the thermal decomposition reaction. For the MBE method, the source materials are heated in different source furnaces and its molecules interact and grow epitaxially on the surface of the substrate in an ultra-high vacuum system at a certain speed and beam ratio. Thus, the precise control of the material size and chemical composition can be achieved, and external doping can be avoided, through the MBE method. For the growth of III–V nanostructures like nanowires (NWs) with MOCVD and MBE methods, the VLS and VSS mechanisms are generally applied depending on the state (liquid or solid) of the seed particles. Specifically, the growth of III–V NWs requires a particle-assisted catalytic process (metal catalysis and self-catalysis) to drive their axial growth. Thus, revealing the dynamic behavior of the droplet during the growth of nanostructure is quite important in understanding their growth mechanisms. In this section, we will simply summarize the synthesis of various III–V nanostructures.

### 2.1. Controlled Synthesis of III–V NWs

**Figure 2:**
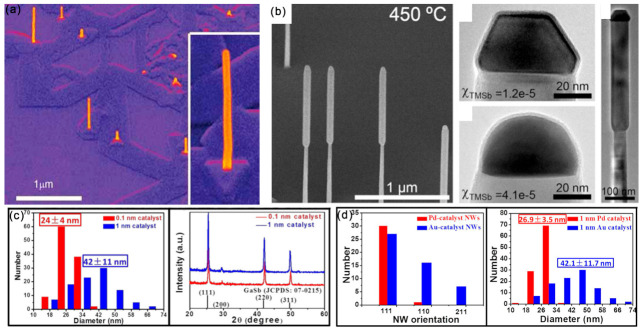
(**a**) The general morphology of InAs NWs on GaAs (111)_B_ substrates. Reproduced with permission [24]. Copyright 2009, Wiley-VCH Verlag GmbH & Co. KGaA, Weinheim. (**b**) SEM and TEM image of GaAs/GaSb nanowires showing the characteristic larger diameter of the GaSb segment. Reproduced with permission [25]. Copyright 2008, Elsevier B.V. (**c**) CMOS-compatible catalyst of Pd was used for the GaSb NW growth. Reproduced with permission [29]. Copyright 2017, American Chemical Society. (**d**) Diameter distribution and crystal structure of the as-prepared GaSb NWs. SEM images of GaSb NWs prepared by using the 0.1 and 1 nm thick Au films as the catalyst. Reproduced with permission [30]. Copyright 2015, American Chemical Society.

Epitaxial III–V semiconductor NWs have attracted much attention due to their potential applications in electronics and optoelectronics [20,21,22,23]. Both “top-down” and “bottom-up” methods have been utilized to synthesis III–V NWs. For the “top-down” method, photolithography and etching are frequently applied to fabricate NWs from their bulk counterpart, while the further scale-down of NWs is limited. For the “bottom-up” process, III–V NWs are epitaxially grown on substrate from atoms and molecules with the VLS and VSS mechanisms, according to the state of seed particles during the growth process. The metal catalyst particle is regarded as one of the most important factors influencing the structure and morphology of NWs. Au catalysts have frequently used in the epitaxial growth of GaAs (Figure 2a), [24] GaSb (Figure 2b) [25] and InAs NWs. Except for the frequently used Au NPs, Pt, Ag and Cu have also been used as seed particles for the epitaxy growth of III–V NWs [26,27,28,29]. The physical properties of III–V NWs are closely related with their growth orientation, phases and diameters; thus, the controlled synthesis of III–V NWs is quite important. 

**Figure 3:**
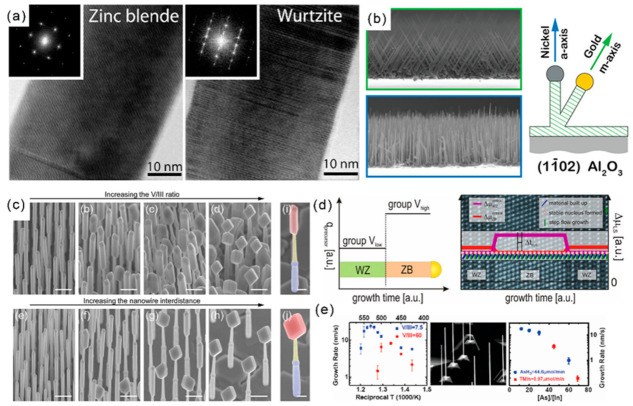
(**a**) HRTEM and the diffraction pattern images of parts of GaP nanowires. Reproduced with permission [31]. Copyright 2009, American Chemical Society. (**b**) Interplay of catalyst composition and epitaxy on the growth orientation of GaN nanowires. Reproduced with permission [32]. Copyright 2014, American Chemical Society. (**c**) The 30° tilted SEM images illustrating the evolution of the InSb nanowire morphology with different parameters. Reproduced with permission [35]. Copyright 2012, American Chemical Society. (**d**) Schematic sketch representing the group V precursor switching sequence vs. growth time for the preparation of WZ-ZB heterostructured III–V NWs using metal-organic vapor-phase epitaxy (MOVPE). Reproduced with permission [36]. Copyright 2013, American Chemical Society. (**e**) Temperature and input V/III ratio-dependent growth rate of InAs NWs on InAs(111)_B_ substrates. Reproduced with permission [37]. Copyright 2007, American Chemical Society.

It should be mentioned that the selection of metal catalysts can influence the NWs’ diameters and orientations. Under the identical conditions and film thickness (1nm), the Pd-catalyzed growth of GaSb NWs presented a smaller diameter and narrower distribution (26.9 ± 3.5 nm) than that of Au-catalyzed NWs (42.1 ± 11.7 nm) (Figure 2c) [29]. The growth directions of the Pd- and Au-catalyzed growth of GaSb NWs were <111> and <211>, respectively. Moreover, by controlling the film thickness of Au, high-quality GaSb NWs, with controllable diameters ranging from 16~70 nm, have been obtained, and their growth directions were found to depend on the NW diameters (Figure 2d) [30]. Importantly, the supersaturation of Au seeds can greatly influence the defect density of III–V NWs [31]. For the low supersaturation Au seeds, the grown GaP NWs present a nearly defect-free ZB structure, while the planar defect density increases with the increasing supersaturation of Au seeds (Figure 3a). The WZ-structured GaP NWs are formed with high supersaturation of Au seeds. Moreover, by modulating the Au/Ni ratio in the bimetallic catalyst, the selective growth of dense and uniform III–V NW arrays along two non-polar directions (Figure 3b) can be achieved [32]. 

The input III/V precursor ratio also plays a key role in controlling the NW mor-phology. Generally, the radial growth rate of a III–V semiconductor increases with the increase of V/III ratio [33,34]. During the growth of InSb NWs on the (111)B InP sub-strate, the morphology of InSb can change from NWs to nanocubes with the increase of the V/III ratio (Figure 3c) [35]. The flux of the source affects the phases of the synthe-sized III–V NWs. Specifically, the V-group flow is lower for the WZ phase and higher for the ZB phase. The single and mixed WZ-ZB heterostructures of GaP, GaAs, InP and InAs NWs with sharp crystal phase interfaces have been successfully prepared by con-trolling the V group flow rate during the synthesis process (Figure 3d) [36]. The growth temperature can influence the growth rate and morphology of NWs (Figure 3e) [37]. It has been found that the proportion of the WZ phase of InAs NWs increased signifi-cantly with the decrease of temperature, providing solutions for the synthesis of high-purity WZ InAs NWs and WZ-ZB superlattices [38]. The influence of catalysts and temperature on the dynamic morphology and phase transition of III–V NWs has been investigated with in situ TEM techniques and will be introduced in the following section.

Except for the epitaxial growth method, III–V NWs can also be synthesized on the SiO2/Si substrate through a solid-source CVD with the VLS mechanism [39]. Due to the incompatibility of Au in the complementary metal oxide semiconductor (CMOS), the practical applications of the Au-catalyzed synthesis of III–V NWs in the large-scale semiconductor field is quite limited. Since the diffusion of Pd into Si can form Pd2Si, which can act as electrical contacts in devices, GaSb NWs compatible with CMOS technology have been synthesized via Pd catalysis (Figure 3e) [29,40]. In addition, the direct growth of III–V NWs on a Si substrate has also been carried out. With the help of a SiO2 nanotube template, the vertical Si-InAs NW tunnel diode has been directly manufactured on (100), (110), (111) and (112) Si wafers [41].

### 2.2. Growth of Other III–V Nanostructures: Quantum Dots (QDs) and Nanosheets

#### 2.2.1. Synthesis of QDs

The synthesis of III–V QDs has recently attracted extensive attention due to their remarkable quantum confinement effect, [42,43] large exciton radius [44], etc. The III–V QDs have shown potential applications in gas sensing [45] and biological detection [46,47,48]. Due to the highly reactive group-V precursors, the synthesis of III–V QDs is quite challenging.

**Figure 4:**
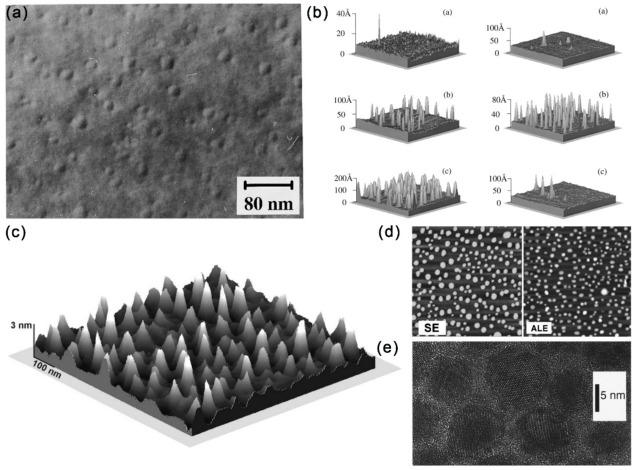
(**a**) TEM image of GaSb/GaAs quantum dots formed with GaSb deposition. Reproduced with permission [49]. Copyright 1995, American Institute of Physics. (**b**) AFM images of InSb grown on GaAs at 400 °C: ~1.5 ML InSb, ~ 2.0 ML InSb and ~ 3.5 ML InSb (left), and AFM images of 2.0 ML InSb grown on GaAs: ~350 °C, ~430 °C and ~460 °C (right). Reproduced with permission [50]. Copyright 1996, American Institute of Physics. (**c**) AFM image of a 0.8 × 0.8 μm^2^ surface area showing the formation of GaAs dots [51]. Copyright 1998, American Institute of Physics. (**d**) AFM images of two samples with nominally 2.5 ML of InSb on InAs. Left: sample grown using SE; Right: sample grown using atomic-layer epitaxy. Reproduced with permission [52]. Copyright 2006, American Institute of Physics. (**e**) Higher resolution TEM image of InAs nanocrystals Reproduced with permission [42]. Copyright 1996, American Institute of Physics.

The strain-induced growth of QDs through the Stranski–Krastanow mechanism with the MBE method has been applied to synthesize InSb, GaSb and AlSb on the GaAs substrate (Figure 4a,b) [49,50]. The lattice mismatch finally leads to the formation of islands after the deposition of planar wetting layers. In contrast to the conventional MBE method, an improved droplet epitaxy method for the preparation of self-organizing GaAs/AlGaAs QDs with high As-flux irradiation and low substrate temperature has been proposed (Figure 4c) [51]. To promote the three-dimensional growth mode, the (1 × 1) AlGaAs surface was alternately exposed to Ga and As sources to obtain GaAs nanocrystals with {111} facets. The transition to a three-dimensional growth mode is attributed to Ga adsorption atoms on the GaAs surface, with excess As at low substrate temperatures. Fariba Hatami et al. has also reported similar growth of InSb and InSb:N QDs on InAs and GaAs substrates (Figure 4d) [52].

Except for the MBE method, the MOCVD method is also frequently applied to synthesis III–V QDs. Specifically, the saturated vapor of the reaction source is brought to the reaction chamber and mixed with other reaction gases when the current-carrying gas passes through the container of the organometal reaction source, and then the chemical reaction occurs on the heated substrate to promote the growth of the film [53]. Moreover, InAs QDs (2.5~6.0 nm) have also been synthesized with the colloidal chemical synthesis method (Figure 4e) through the reaction of InCl_3_ and As[Si(CH_3_)_3_]_3_ [42,54,55,56]. The synthesized InAs QDs are of comparable size to InAs dots prepared by MBE strain-induced islanding methods, while the colloidal chemical synthesized InAs QDs are spherical, and MBE dots are square pyramidal.

#### 2.2.2. Two-Dimensional (2D) III–V Materials

**Figure 5:**
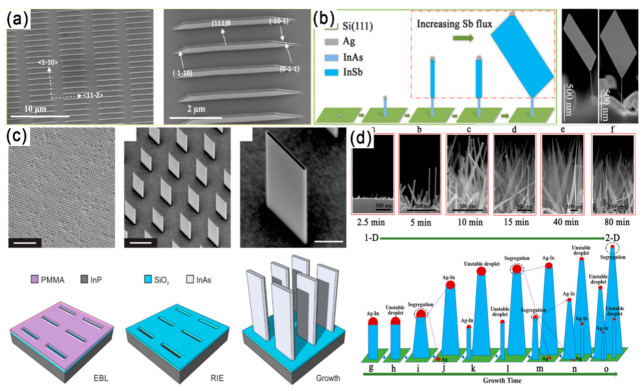
(**a**) Macroscopic SEM image of nanosheet arrays and a single nanosheet. The inclined surfaces of the nanosheets are three {110} planes [61]. Copyright 2013, American Chemical Society. (**b**) Diagram of the growth process and SEM images of the InSb nanosheets. Reproduced with permission [62]. Copyright 2016, American Chemical Society. (**c**) Templated growth of 2D InAs nanofin structures. Reproduced with permission [63]. Copyright 2019, American Chemical Society. (**d**) Experimental results of the InAs grown under the same indium-rich conditions with different growth times and a schematic demonstration of the catalyst alloy segregation process for the dimensional evolution of InAs from 1-D to 2-D. Reproduced with permission [28]. Copyright 2019, American Chemical Society.

2D III–V materials are generally grown by MBE or MOCVD methods [57,58,59]. Among all III–V semiconductors, InSb has the narrowest band gap, the smallest effective mass of carriers and the highest mobility of carriers, which makes it have great potential in infrared detection and spintronics. Recently, a heterojunction structure consisting of NWs and nanosheets has been successfully synthesized. Maria de la Mata et al. has reported the synthesis of vertical InSb nanosails grown epitaxially from an InAs “mast” acting as a stem on an InP (111)_B_ substrate, with a thickness controlled by the seed particle, two large atomically flat (110) surfaces and a highly faceted geometry [60]. The nanosail crystal structure is pure ZB with only a single isolated twin, which drives the crystal to change its geometry and expand to create the 2D morphology of InSb. The twin-free GaAs nanosheets have been fabricated on a GaAs (111)_B_ substrate through the selective area growth method. The synthesized GaAs nanosheets possess five equivalent (110) surfaces: two vertical $\left(1\bar{1}0\right)$ surfaces and three inclined $\left(\bar{1}\bar{1}0\right)$ surfaces (Figure 5a) [61].

Later, the growth of free-standing InSb nanosheets on 1D InAs NWs through MBE on a Si substrate is proposed (Figure 5b) [62]. The morphology and size of free-standing InSb nanosheets can be controlled in the approach by tailoring the Sb/In beam equivalent pressure ratio and InSb growth time. Through this growth mechanism, pure ZB single crystal InSb nanosheets with length and width up to several microns and thickness up to 10 nm were synthesized. The above mentioned InSb nanosheets are epitaxy on InAs stems. In 2019, Sasa Gazibegovic et al. grew InSb nanosheets directly from a InSb substrate platform without any foreign stem formation [64]. With a SiO_x_ dielectric template fabricated by a mask transfer process by the plasma-enhanced CVD, the rectangular InAs nanofins with definite length, width and height can be synthesized at the rectangular opening using the MOCVD method (Figure 5c) [63]. By controlling the In flux, the morphology of InAs can be adjusted from 1D NWs to 2D nanosheets (Figure 5d) [28].

By comparing the growth methods of III–V NWs and nanosheets, it can be found that the lateral epitaxial growth is mainly inhibited to achieve controllable longitudinal synthesis during the growth of 1D NWs. The thickness of Au catalytic film was usually reduced to prevent the uncontrolled radial growth of NWs [30]. In contrast, the growth of nanosheets requires both lateral and longitudinal epitaxial growth and is usually accompanied by the growth of related NWs [64].

## 3. Growth Mechanisms of III–V NWs Revealed by In Situ Techniques

Since NW growth can be affected by parameters like the morphology of seed particles, temperature, pressure, etc., identifying their specific influence can provide guidance on the high-quality material preparation. Moreover, a clear understanding of the NW growth mechanism is indispensable for well-controlled growth of structures with desired properties. However, the traditional post-growth method can hardly accurately obtain important growth parameters. The dynamic composition, morphology and structural evolution of the catalyst/droplet NPs are quite important to understanding the phase selection, growth-direction switching and defects formation.

### 3.1. Role of the Catalysts/Droplets

**Figure 6:**
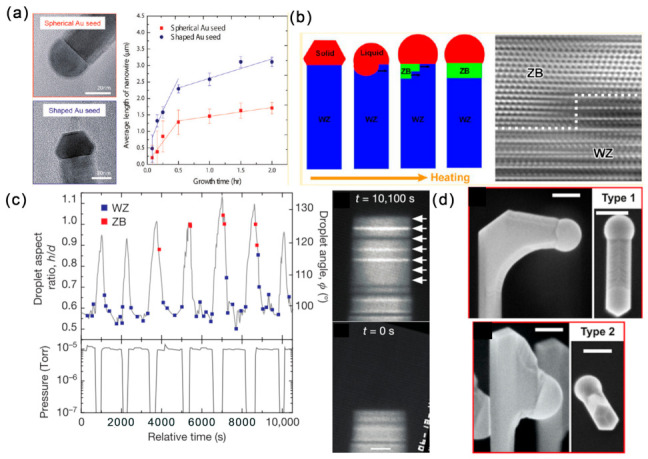
Influence of droplet NPs on the growth of III–V NWs. (**a**) Relationship between the average length and growth time of InAs NWs grown with spherical and shaped Au seed particles. Reproduced with permission [67]. Copyright 2012, American Chemical Society. (**b**) Role of Au particles in tailoring the nucleation and phases of InAs NWs at the liquid/solid interface. Reproduced with permission [68]. Copyright 2013, American Chemical Society. (**c**) Growth of a WZ GaAs NW containing multiple narrow ZB by controlling the AsH_3_ pressure and the droplet morphology. Reproduced with permission [69]. Copyright 2016, Macmillan Publishers Limited. (**d**) Side- and top-view images of type 1 horizontal and type 2 downward-grown self-catalyzed GaAs NWs. Reproduced with permission [70]. Copyright 2019, American Chemical Society.

Taking advantage of the in situ heating holders used in the environmental TEM, the composition of the Au catalyst during the growth of GaAs NWs was monitored as they grow. The Ga content in the catalyst was found to increase with both temperature and Ga precursor flux during the Au-seeded GaAs NW growth by the VLS method [65,66]. The initial seed particle morphology can greatly influence the NW growth process. By comparing the InAs NW growth with spherical and faceted Au NPs, Pin Ann Lin et al. found that the shaped Au NPs partially melted during growth and that the surface remained solid and faceted (Figure 6a). The facets of Au NPs can facilitate the adsorption of In vapor and form a thicker In shell, leading to the enhanced growth kinetics of InAs NWs compared with the spherical Au NPs [67].

In contrary to bulk materials, typical ZB-structure III–V semiconductors form nanowires in the WZ structure as well as the ZB structure. Despite the growth rate, seed particles also play important roles in tailoring their nucleation and phases. The Au-catalyzed InAs NWs grown by MOVPE with the VLS mechanism are generally dominated by WZ phase with SFs and ZB phase. With the joule heating realized by the nanofactory holder, the Au catalyst can be molten by controlling the electrical signals. The liquid Au NP is observed to flow and dissolve the InAs NW, with the WZ phase and the WZ to ZB phase transition occurring in the liquid/solid interface or liquid/solid/amorphous carbon triple point (Figure 6b) [68].

For III–V NWs, the structure switching between the ZB and WZ phase is important for the electronic and photonic applications of related heterostructures. By comparing the structure and dynamics differences during ZB and WZ GaAs nanowire growth, the geometry of the catalyst droplet is found as the key parameter in determining structure by influencing the nanowire edge morphology [69]. The growth of WZ GaAs proceeds by step flows across the droplet/NW interface and is limited by the arrival and incorporation of As. However, during the growth of ZB GaAs, the droplet/NW interface presents an oscillating geometry at the solid/liquid/vapor trijunction, leading to a rapid bilayer flow across the growth interface. Moreover, the droplet geometry can be controlled by the V/III ratio. Specifically, the droplet volume increases/decreases with decreasing/increasing AsH_3_ pressure, which is driven by the addition or subtraction of Ga. Moreover, there exists a direct correlation between crystal switch and droplet dimensions (volume, aspect ratio and angle) controlled by the V/III ratio (Figure 6c). Recently, Federico Panciera et al. dynamically monitoring the self-catalyzed VLS growth of GaAs and GaSb NWs through MBE inside a TEM. They found that the appearance of ZB or WZ phase is controlled by the droplet-NW contact angle: small (<100°) and large (>125°) contact angles for ZB phase, while pure ZB phase can be obtained for the intermediate contact angles. Importantly, the contact angle can be finely tuned by changing the III/V fluxes [71].

With in situ HRTEM techniques, the phase transition mechanism between the ZB and WZ phase of III–V nanostructures has been dynamically revealed. He Zheng et al. showed that the WZ to ZB phase transition in InAs NWs is a solid-to-solid phase transition. Dynamic observations further confirmed that this phase transition occurred through gliding of sharp steps with Shockley partial dislocations [68]. Each step was six (111) atomic layers high, and the step migrated without any mechanical stress applied. By investigating the WZ to ZB phase transition at the catalyst/NW interface, it is found that the phase transition took place between 300 and 350 °C. Importantly, the nucleation site of each new layer of InAs and catalyst surface energy plays a decisive role in the growth of the ZB structure [72].

### 3.2. Switching of NW Growth Direction

In the design of three-dimensional NW-based structures, the interconnected NWs have shown their possible applications in optics and photonics [73,74]. Therefore, controlling the NW growth direction of self-catalyzed NWs is quite necessary, and this is closely related with the catalyst droplets. For self-catalyzed GaAs NWs, switching the NW growth direction can be achieved by annealing the vertically grown NW at the growth temperature without any fluxes [70]. This growth interruption led to the droplet-NW interface reshaping: droplets falling toward one of the (110) side facets, and the resumed NW growth is perpendicular to their initial growth direction (Type 1) or slightly downward (Type 2) (Figure 6d). The yield of the type 1 and 2 structures is controlled by the growth parameters of NW density, annealing time, V/III ratio and diameter, suggesting that the growth interface reshaping is caused by the interplay of the surface energetics and kinetics. For the self-catalyzed InAs(Sb) NWs, annealing can realize the formation and manipulation of In droplets, which can be used to initiate growth in different crystalline directions [75]. It should be mentioned that the position of the In droplet can result in the linear or L-shaped nanostructures.

Moreover, catalyst NPs can experience reconfiguration during the joint growth of III–V NWs and can affect the subsequent NW growth in turn. In the growth of interconnect pairs of InAs NWs, due to the doubled volume of Au NPs and reduced Au-InAs interface upon coalescence, the contact angle of Au-InAs will increase, which can result in the catalyst depinning. The droplet configuration pinned across three facets of InAs NW can lead to the subsequent sidewall crawling NW growth [73].

## 4. Dynamic Structural Evolution of III–V NWs under External Field

Clarifying the structural evolution of III–V group nanomaterials under external stimulus is fundamentally important for their growth, and design, fabrication and applications of related electronic devices. Due to the small size of III–V group nanomaterials, in situ techniques are frequently used to reveal their microscopic mechanism and establish a property–structure relationship by taking advantage of the high special resolution of TEM.

### 4.1. Dissolution/Sublimation of III–V NWs

**Figure 7:**
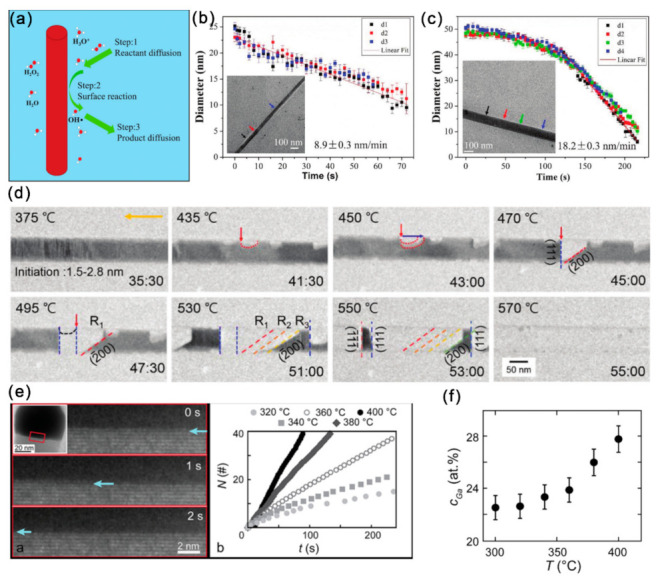
(**a**) Schematic diagram presenting the dissolution procedure of InAs NWs in the radiolytic water. The diameter of (**b**) an MBE-grown and (**c**) a MOCVD-grown InAs NWs vs. irradiation time. Reproduced with permission [13]. Copyright 2018, The Royal Society of Chemistry. (**d**) A series of TEM images captured during the in situ heating process. Reproduced with permission [76]. Copyright 2019, The Royal Society of Chemistry. (**e**) TEM image series showing the layer-by-layer removal of GaAs at the droplet/NW interface. The extracted cumulative number of bilayers (N) removed is also presented as a function of time (t). (**f**) Ga content of the droplet particle as a function of temperature, presenting a monotonic increase of Ga with temperature. Reproduced with permission [77]. Copyright 2019, American Chemical Society.

Understanding the structural transformation of III–V nanostructures at high temperatures and in chemical environments is quite important for both science and technology in many fields. For their applications involving a liquid phase process, the recently developed liquid cell technology provides us an effective approach to in situ observe the liquid phase reactions through TEM. The III–V NWs have shown promising applications in high-performance electronic devices, while their surface-native oxide layer can greatly affect their physical properties, and wet-chemical etching has been frequently applied to remove the oxide layer and passivate the surface. Therefore, revealing the dissolution kinetics of III–V NWs is key for the precise control of the etching process. The high-energy electron beam irradiation of water can excite reducing and oxidizing radicals, which can etch InAs NWs (Figure 7a) [13]. The InAs NWs in the radiolytic deionized water presented an unexpected constant dissolution rate (Figure 7b). It should be mentioned that the MOCVD-grown InAs NWs presented delayed dissolution (Figure 7c) with higher dissolution rates than the MBE-grown NWs. This could be caused by the unintentional carbon doping and dense stacking faults of MOCVD NWs. The dissolution kinetics of constant speed InAs NW dissolution in radiolytic water is found to be surface-reaction limited dissolution, and the reaction activation energy should be constant during the whole dissolution process.

The sublimation behavior of semiconductor materials can provide valuable information for growth direction controlling, surface conditions and device applications, while the sublimation dynamics can hardly be revealed through the traditional technologies like mass spectrometry and total weight loss measurements. In binary compound semiconductors like InAs and GaAs, the polarity plays important roles in the etching rate, growth rate, and their sublimation behavior. With the in situ heating chips, it has been found that the sublimation of InAs NWs was initiated at the edge and corner regions and propagated by forming specific atomic structures. Moreover, InAs NWs presented anisotropic atomistic evolution during the sublimation process: decomposition along the [111] direction occurred with the formation of (200) planes, while that along the $\left[\bar{1}\bar{1}\bar{1}\right]$ direction occurred with the formation of the relatively large steps on the (111) planes (Figure 7d). Theoretical calculations further predicated that the In-terminated planes were relatively stable than the As-terminated planes. When InAs NWs were heated in an H_2_ atmosphere in an ETEM, it can be found that the morphology of the Au-containing NPs changed dynamically and the dissolution of the InAs occurred at the catalyst-NW interface with crystallites formed adjacent to the NWs [78].

The stability of NWs in contact with the droplet particles and the particle composition evolution with temperature is another key issue related to the growth of III–V NWs. Very recently, the droplet-assisted decomposition of GaAs was observed, and the decomposition rate was controlled by the amount of Ga in the droplet [77]. Specifically, during vacuum annealing between 300~420 °C, the GaAs facet in contact with the droplet was removed through a thermally activated bilayer-by-bilayer manner (Figure 7e). The Ga content in the droplet particle remained constant at any given temperatures and increased with increasing temperature (Figure 7f). For self-catalyzed GaAs NWs, the atomistic evolution at the interface between Ga droplets and GaAs NWs during heating was also quite necessary to understand the complex growth behaviors. The Ga droplet changed morphology, and the formation and destruction of a few monolayer-thick ZB GaAs can be observed at the Ga/GaAs interface at ~200 °C. When temperature was increased above 300 °C, the volume of the Ga droplet decreased rapidly, and thr fast growth of ZB GaAs structures can be observed in the droplet. Finally, the decomposition of GaAs began at the WZ region and propagated to the ZB region at ~600 °C [79].

### 4.2. Contact Formation Mechanism

Ohmic source/drain contacts formation is one of the major challenges associated with nanodevices. The low contact resistivity with ohmic interfaces is desired for reduced parasitic resistances. The phase transformation along with the metal-semiconductor compound contacts to semiconductor channels is an important aspect of nanodevices. It has been reported that the InSb segment in the high-quality InAs/InSb heterostructure NWs with [111]_B_ direction can form near-perfect ohmic contact with a Ni/Au electrode [80]. Although the Fermi level of InAs pins in the conduction band, it has been experimentally observed that the contact resistivity of n-InAs can be decreased with the increase of the concentration of InAs active carriers. When the active carriers exceeded a certain amount, the ultra-low resistance ohmic contact between InAs and (Ti/Au/Ni) metal electrodes could be effectively achieved [81]. The contact resistance of the material can be greatly changed by changing the metal electrode in contact. It should be mentioned that Ti/Ni electrodes can provide ohmic contact with InAs nanowires at very low resistivity and have become the ideal substitute for Ti/Au electrodes commonly used for InAs nanowires [82]. Similar metal electrode systems such as Ti/Pt/Au and Ti/Ni/Au have been reported to achieve good ohmic contact with III–V materials [83,84].

**Figure 8:**
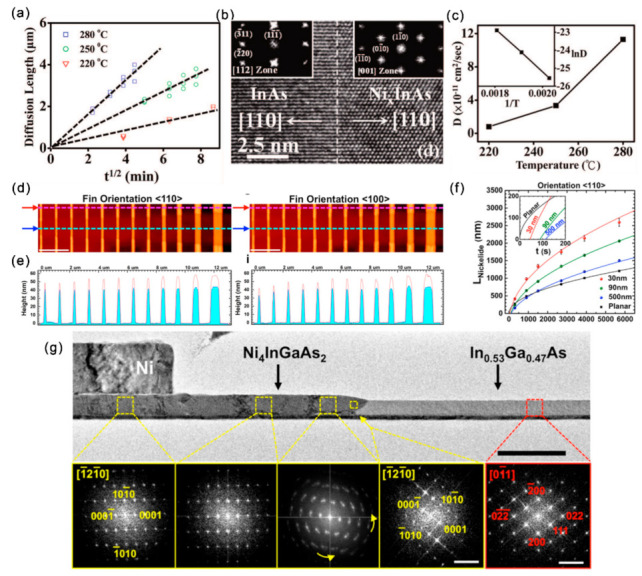
(**a**) Ni diffusion length vs. t^1/2^ at diffusion temperatures of 220, 250 and 280 °C (**b**) HRTEM image showing the InAs/Ni_x_InAs interface. (**c**) The diffusivity of Ni in InAs as a function of temperature. Reproduced with permission [88]. Copyright 2008, American Chemical Society. (**d**) AFM topography plots of the nickelide-InGaAs Fin channels. Scale bars are 2 μm. (**e**) The height profiles for nickelide segments and nonreacted InGaAs segments correspond to the line cuts in (**d**). (**f**) The length of nickelide segments vs. annealing time at 250 °C for <110> Fin orientations. (**g**) TEM image of the [110]-oriented Fin with nickelide formation. Reproduced with permission [91]. Copyright 2015, American Chemical Society.

Thermal annealing is generally used to improve the contact quality. The structure of the alloyed contacts can alter the contact resistivity, influence the contact reliability and introducing strains, which can greatly affect the band structure and carrier mobility of III–V channel materials [85,86,87]. Thus, characterizing the metal/III–V contact formation, interface structure and composition is quite necessary. The Ni_x_InAs/InAs/Ni_x_InAs heterojunctions can be formed through a solid source reaction of Ni with InAs when annealing the Ni-contacted InAs at temperatures of 220~300 °C [88]. There exists a linear relationship between the diffusion length (*X*) and *t*^1/2^ (t represents annealing time) for each annealing temperature (220, 250, 280 °C) (Figure 8a), suggesting that the Ni/InAs alloying process is limited by the Ni diffusion into InAs. This *t*^1/2^ dependence has also been observed in nickelide formation in InGaAs Fins and GaAs thin films. The solid reaction of Ni with InAs occurs led to the formation of sharp epitaxial interfaces (Figure 8b). According to the diffusion limited model of *X = (Dt)*^1/2^, the diffusivity *D* of Ni into InAs are calculated to be 8 × 10^−12^, 3.35 × 10^−11^ and 1.13 × 10^−10^ cm^2^/s at 220, 250 and 280 °C, respectively (Figure 8c). Based on the Arrhenius relationship, *D*∝*e^−Ea/kT^*, the activation energy extracted from the temperature-dependent diffusivity is 1.04 eV/atom.

The crystal orientation, size and phase can play dominant roles in device performance [89,90]. Therefore, revealing the size and orientation effects on the solid-state reaction kinetics and the structure of the metallic contact with III–V nanostructures is also quite important. Along with the formation of nickelide (Ni-InGaAs compound), the In_0.53_Ga_0.47_As Fin channel presented a 33% ± 5% height increase with negligible lateral expansion with a flatter interface for <110> oriented Fins compared to <100> ones (Figure 8d,e) [91]. By investigation the influence of time, temperature and geometrical factors of InGaAs Fin channels, Renjie Chen et al. revealed the size-dependent Ni surface diffusion dominant process during the nickelide formation, which gradually departed to volume diffusion with the increase of Fin width. Due to the surface-diffusion limited reaction, smaller Fins experience faster incubation times for the formation of nickelide (Figure 8f). The formed nickelide was characterized as Ni_4_InGaAs_2_ with Ni_4_InGaAs_2_ $\left[\bar{1}2\bar{1}0\right]$//In_0.53_Ga_0.47_As $\left[0\bar{1}1\right]$ and Ni_4_InGaAs_2_(0001)//In_0.53_Ga_0.47_As (111) with a peculiar rotation of the Ni_4_InGaAs_2_ [0001] axis away from the nickelide/InGaAs interface (Figure 8g).

**Figure 9:**
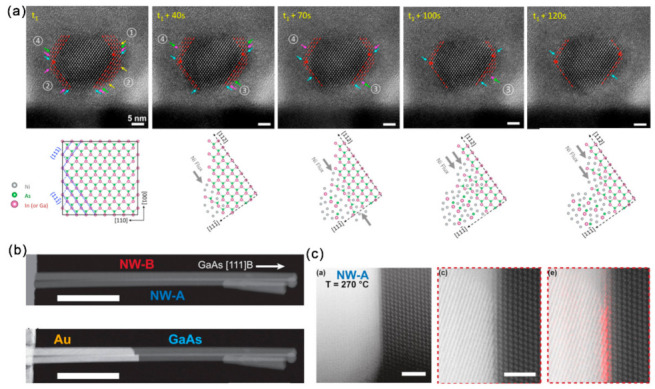
(**a**) The ledge formation and movement mechanisms at atomic resolution and the proposed mechanism of ledge movement on the $\left(11\bar{1}\right)$ plane along [112] or $\left[\bar{1}\bar{1}\bar{2}\right]$ directions. Reproduced with permission [92]. Copyright 2017, American Chemical Society. (**b**) HAADF-STEM images presenting the GaAs NWs before (upper) and after (bottom) partial heat-induced replacement by Au. (**c**) HAADF-HRSTEM images showing the interface of Au–GaAs at elevated temperatures. Reproduced with permission [12]. Copyright 2016, American Chemical Society.

With the development of in situ techniques, contact formation dynamics can be revealed from the atomic level. Expect for the solid-state reaction occurring along the channel length direction, the dynamic reaction in the cross section, which can control the final phase and crystalline orientations of the horizontal reactions, remains unclear. A solid-state amorphization process was observed for the In_0.53_Ga_0.47_As NW cross section [92]. In situ characterization revealed that the formation of amorphous nickelide (Ni_x_In_0.53_Ga_0.47_As) was realized through a layer-by-layer manner proceeding by ledge movements on (111) facets along <112> directions (Figure 9a). The Ni- In_0.53_Ga_0.47_As interface can greatly influence the reaction kinetics. Through an annealing above 375 °C, the amorphous Ni_x_In_0.53_Ga_0.47_As can further recrystallize to single crystalline Ni_2_In_0.53_Ga_0.47_As through thr additional incorporation of Ni from the contact. The solid-state replacement has also been reported for Au-contacted GaAs.

Although Au and Au-based alloys are frequently used in forming ohmic contacts with the Ga_x_Al_1-x_As system, the underlying reactions during this alloying process remains unclear. Earlier studies on GaAs-Au thin film revealed the evaporation of As with temperature, time and Au thickness [93,94]. During the reaction of GaAs with Au film, GaAs decomposes to form a Au-Ga solid solution and the diffusion of Ga in Au is the rate-limiting step of this dissolution. It has also been shown that the interface condition can severely influence the rate and extent of this reaction [95,96]. Importantly, the slowest reaction direction in the thin film system is reported to be normal to the (111) GaAs plane with a preference for forming an As-terminated (111) surface. In the in situ heating stage, the dynamics of the thermally induced replacement of GaAs NWs by Au has been tracked [12]. For the endless reservoir configuration (Figure 9b), the metal phase remained solid when it was connected to the Au reservoir. Although there presents no fixed relationship between the crystallographic orientation of Au and GaAs phases in the heat-treated Au-GaAs NWs, their relative orientation seems to influence the reaction rate and its temperature dependence. It is also found that the replacement rate can be affected by twining in the Au phase. In situ dynamic observations showed that the Au–GaAs interface mainly followed the GaAs (111) plane and the replacement occurred one GaAs bilayer at a time (Figure 9c). In contrast, for the limited Au reservoir, a decrease in the reaction rate was observed as the Ga content in the Au reservoir increased. The metal phase can become liquid in this case.

### 4.3. Energy Storage Mechanism

Recently, III–V nanomaterials like InSb have become appealing anode candidate due to their good electronic conductivity. The commercially available InSb has been reported to exhibit an initial specific charge capacity of 680.9 mA h g^−1^ and maintained 78.6% of the initial charge capacity after 80 cycles for a lithium-ion battery. Ex situ characterization showed that the good electrochemical properties are caused by the high structural stability and electrochemical reversibility [97]. Hiroyuki et al. further revealed the reaction mechanism for a Na-ion battery [98]. Firstly, the InSb electrode exhibited a good cyclability with the reversible capacities of over 400 mA h g^−1^ for 250 cycles. They found that the phase separation occurred in the first sodiation to form the nanostructure in which In nanoparticles were dispersed in the Sb matrix. During the desodiation reaction, the above separated phases of In and Sb didn’t recover to the original InSb phase. As an important III–V group semiconductor, InAs has a relatively high theoretical capacity of 1034 mA h g^−1^ for Li_13_In_3_ and Li_3_As. The high electron mobility, narrow direct bandgap endowed InAs can possess much higher intrinsic electronic conductivity than the Ge and Si anode. With the in situ TEM technique, [90,99] we investigated the ionic transport properties, structural evolution, cyclability, and reaction mechanisms of the lithiation/delithiation process of single InAs NWs [13]. A two-stage lithiation behavior including the insertion of a Li-ion into the NW lattice and the conversion and alloying processes induced the formation of Li_3_As and Li_x_In. InAs NWs also show a fast reaction speed of ~275 nm/s and a high Li-ion diffusion coefficient of 2.49 × 10^−8^ cm^2^/s. A relatively small volume expansion of 157% can be observed in full lithiated NWs. Length contraction and the dealloying process of Li_x_In take place can be observed during the delithiation process. Our in situ investigations indicates that the insertion/extraction of Li-ion and the alloying/dealloying of Li_x_In are reversible and that InAs NWs may have good cyclability.

## 5. Structural–Dependent–Physical Properties of III–V NWs

Generally, synthesis conditions determine the morphology and structure of materials, which can greatly influence their physical properties [100]. In this section, we will introduce how the structure parameters, such as orientation, phase, size, defect and doping, affect their physical properties.

### 5.1. Electronic Properties

**Figure 10:**
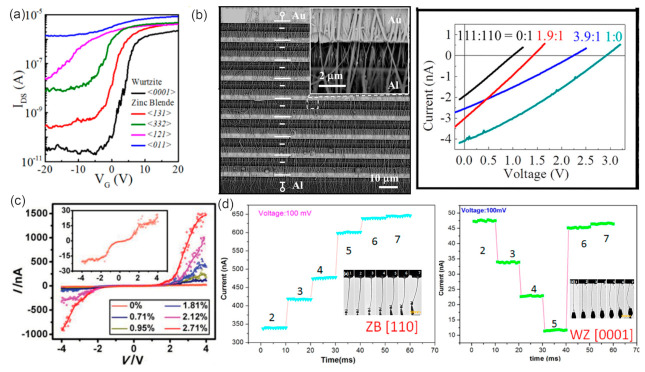
(**a**) I_DS_-V_G_ characteristics of the back-gated NW FETs for five different oriented InAs NWs in logarithmic coordinates. Reproduced with permission [104]. Copyright 2016, American Chemical Society. (**b**) Output performance of the cells fabricated with different mixing ratios of <111>- and <110>-oriented NWs. Reproduced with permission [107]. Copyright 2016, American Chemical Society. (**c**) Electromechanical measurement of [0001]-grown WZ InAs NWs and the I-V curves of the NW measured at various tensile strains. Reproduced with permission [116]. Copyright 2015, WILEY-VCH Verlag GmbH & Co. KGaA, Weinheim. (**d**) Variation of the current with increasing deformation of the ZB <110> InAs NW and WZ <0001> InAs NW under a bias of 0.1 V. Reproduced with permission [117]. Copyright 2016, American Chemical Society.

NWs with different growth orientations can present distinct effective mass and carrier mobility, [101,102,103] revealing that the optimal effective mass and carrier mobility in a specific direction is of great importance for improving the performance of III–V NW-based electronics. For the electronic properties of the field-effect transistors based on InAs NWs, it has been reported that the subthreshold swing increases while threshold voltage, on–off ratio and the effective barrier height at the off-state decrease one by one in the sequence of WZ <0001>, ZB <131>, ZB <332>, ZB <121> and ZB <011> (Figure 10a) [104]. GaAs NWs have shown excellent photovoltaic properties and presented promising applications in solar cells [105]. The GaAs NWs with the WZ phase present a bandgap of 1.444 ± 0.001 eV, which is 20 meV more than that of its ZB phase (Figure 9b) [106]. The open-circuit voltages *V_oc_* of GaAs NW arrays with pure <111> and <110> orientation had been reported to be 0.33V and 0.12V, respectively, while *V_oc_* for NW array with mixed orientations presented values between 0.12 and 0.33V (Figure 10b) [107].

When the dimensions of nanomaterials are reduced down to nanoscale, size can play an important role in modulating their physical properties [108]. Firstly, the diameter of NWs can greatly affect their electronic properties [109,110]. The field-effect mobility of InAs NWs has been found to linearly increase with radius for 7–18 nm [111]. Moreover, the nearly linear decrease of the measured conductivity with a reduced GaAs NW diameter from 120 nm to 20nm has also been revealed [112]. It has been found that the carrier lifetimes in GaAs NWs are very sensitive to NW diameter, and that short carrier lifetimes (1–5 ps) can be attributed to the high surface recombination velocity at the GaAs surface [113]. In contrast, the carrier lifetimes in InP NWs were greater than 1 ns regardless of the NW diameter. The field-effect mobility of InSb NWs presents strong diameter-dependence. The InSb NWs in the low-diameter range possess very high resistivity and low extracted mobility, whereas thicker NWs show improved values [114]. Various defects are often inadvertently introduced in the growth process of III–V semiconductors. The uncontrolled formation of crystal defects can lead to unacceptable device characteristics and variability based on III–V semiconductors. A sharp decrease of stacking-fault (SF) density was observed in the InAs1-xSbx NW with increasing Sb content. The decreased SF density finally leads to a significant increase in the field-effect mobility [115]. It should be mentioned that mobility was not significantly affected by the existence of twins.

### 5.2. Electromechanical Properties

It is known that strain has an important role in the electronic and optical properties of NWs. Understanding the electromechanical properties and revealing the modulation of structure and defects are essential for the design and applications of nanodevices [118,119,120]. The non-centrosymmetry of WZ structure and the narrow bandgap indicates that III–V NWs might be promising materials exhibiting the piezotronic effect in high frequency electronics and piezo-phototronic effect in near infrared wavelength [121,122]. We have in situ investigated the electromechanical properties of individual InAs NWs and established their relationship with crystal structures and defects [116]. When a [0001]-oriented WZ InAs NW is stretched along its axis, the relative displacement of In^3+^ with respect to As^3−^ results in net ionic charges at its two ends and piezoelectric field along the [0001] direction will be built. The piezoelectric file can lead to the bending of energy bands and result in asymmetric energy barriers for the electrons injecting from the two probes. Besides, the resistance of InAs NWs can also be changed by the piezoresistive effect. Due to the coexistence of piezoelectric and piezoresistive effects, the <0001>-oriented WZ InAs NWs presented remarkable and asymmetric electric conductance increase for positive and negative bias voltages under tensile strains (GF reaches 2820) (Figure 10c) [116].

However, single-crystalline $\langle 11\bar{2}0\rangle$-oriented WZ NWs and <011>-, <103>- and <211>-oriented ZB NWs exhibited negligible electromechanical property. Moreover, the piezoelectric effect in single-crystalline <0001>-orientated WZ NWs can be significantly suppressed by SFs. Besides, WZ- and ZB-structured InAs NWs show distinct electromechanical responses: the conductance increases under compressive deformation processes of the ZB <110> NW, while it decreases at the beginning and then increases with a similar deformation process for the WZ <0001> NW (Figure 10d) [117]. The electromechanical properties of the core-shell InAs/In_0.6_Ga_0.4_As NW differ from the bare InAs NWs. The piezoresistance coefficient of the core-shell NW is about two orders of magnitude larger than the bulk InAs and InAs NWs. The change in resistivity in the core−shell NW can be largely attributed to a reduction in band gap that increases the conduction electron concentration in InAs [123].

### 5.3. Mechanical Properties

**Figure 11:**
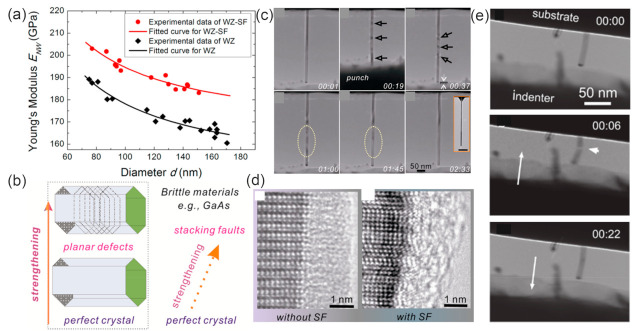
(**a**) The effective Young’s modulus of WZ and WZ-SF GaAs NWs as a function of diameter. Reproduced with permission [15]. Copyright 2016, American Chemical Society. (**b**) The strengthening in brittle materials through defect engineering (via the insertion of planar defects). Reproduced with permission [129]. Copyright 2013, American Chemical Society. (**c**) A series of TEM images showing the unelastic behavior of GaAs. Bending contours are marked with arrows. (**d**) Typical HRTEM images of the interface in a NW without and with stacking faults. Reproduced with permission [131]. Copyright 2013, American Chemical Society. (**e**) TEM images showing the fracture and self-healing of a GaAs NW when a compression force was applied and retracted. Reproduced with permission [132]. Copyright 2011, American Chemical Society.

With in situ static bending and dynamic resonance methods, the Young’s modulus of InAs NWs with diameter of 93–103 nm in the [0001] direction is measured to be 43.5 GPa, much lower than that of cubic bulk InAs in the [111] direction (97 GPa) [124]. Theoretical calculations have shown that the Young’s modulus of the [111] ZB phase InAs NWs decrease while the Poisson’s ratio increases with decreasing NW diameters [125]. By investigating the mechanical parameters of InAs NWs grown by MBE and MOCVD methods, a general trend of fracture strength increase with NW volume decrease is observed for both types of NWs [126], indicating that the fracture of InAs NWs is closely related to their volume, with the number of the flaws responsible for NW fracture increasing with NW volume increase. Due to the increasing volume fraction of the native oxide shell, the Young’s moduli of both WZ and WZ-SF GaAs NWs were reported to increase with decreasing diameter (Figure 11a) [15]. In situ mechanical resonance tests showed that the quality factors of the ZB GaAs NWs was governed by surface effects increasing linearly with the NW radius. The pristine ZB GaAs NWs were shown to have a strong size effect below radii of 100 nm [127]. The in situ resonance and compression tests all showed that the effective Young’s moduli of pristine ZB and WZ GaAs NWs were similar. For GaP NWs grown by the MOVPE (ZB with high density of twinning defects) and MBE methods (WZ with high density of SFs), Prokhor A Alekseev et al. reported a similar Young’s modulus (155 ± 20 GPa and 157 ± 20 GPa) or both types of NWs with the AFM method. Thus, they concluded that crystal structure (WZ/ZB) and crystal defects on the Young’s modulus of GaP NWs can be negligible [128].

Stacking faults (SFs) are commonly existing defects in III–V NWs and can greatly influence a variety of physical properties. Therefore, understanding the effect of SFs on mechanical behavior is quite important for applications in nanodevices, especially flexible devices. It has been reported that the mechanical properties of III–V NWs can be strengthened through defect engineering. Quantitative in situ testing showed that the fracture stress of GaAs NWs with ZB–SF structure (wurtzite with high-density stacking faults) present unusually high fracture stress, more than than pure ZB- or WZ-structured NWs (9.0 vs. 5.4 and 6.2 GPa) (Figure 11b) [129]. The presence of high-density SFs can lead to the formation of short polytypic segments, resulting in the improvement of Young’s modulus. This stiffening effect of SFs is attributed to the change in the interatomic bonding configuration at the SFs [15]. Thus, the SFs can be used to tailor the mechanical properties of III–V NWs. By investigating the fracture behavior of the twining superlattice InP NWs with the push-to-pull method, it can be found that the cracking occurred along their twin boundary interface and that no inelastic deformation was observed [130].

Materials subjected to external stress larger than their yield stresses can experience elastic deformation, followed by plastic deformation. With the help of the in situ TEM nanocompression technique, the Young’s modulus of MOCVD-grown GaAs NWs was found to increase significantly with decreasing NW diameter [133]. Importantly, the combined elastic and plastic deformation exists in GaAs NWs with very small diameters (<25 nm), and the plastic deformation occurred through dislocation activities [134]. The GaAs NWs also exhibit remarkable anelasticity behavior, which can hardly be presented in brittle single-crystal materials (Figure 11c) [131]. It should be mentioned that only NWs with small diameters exhibit anelastic behavior. Besides, NWs with stacking faults have smaller anelastic strain and shorter recovery time than NWs without stacking faults. These phenomena indicates that the amorphous/crystalline interface of the NWs plays a critical role on the anelasticity behavior of the GaAs NW (Figure 11d).

Interestingly, a spontaneous self-healing process was observed in GaAs NWs. The partially fractured GaAs NW can restore the single crystal structure immediately after the release of external compressive force (Figure 11e) [132]. Molecular dynamic simulations showed that this self-healing behavior depends on lateral dimensions and the number of healing cycles [135]. A sufficiently smooth fracture surfaces and strong surface attraction should be preconditions for the self-healing of fractured GaAs NWs. The surface attraction due to the electrostatic interaction in the nearfield region contributes to the Ga-As rebonding. A large lateral dimension and repeated fracture increase the roughness of the fractured surface and hinder the self-healing of GaAs NWs. Additionally, since the thermal motion of surface atoms can increase the mismatch between two fracture surfaces, the healing efficiency can be greatly influenced by the temperature. Annealing-treatment-induced atomic diffusion can help to eliminate the mismatch and enhances healing efficiency.

### 5.4. Optoelectronic Properties

Due to their unique dimension, finite size effect and excellent surface area to volume ratio, NWs have been proven to have great potential as active materials for photovoltaic devices such as solar cells. In particular, III–V material NWs are widely used in high-performance photodetector configurations due to their high electron mobility, appropriate direct bandgap, and stable stability at room temperature [1,2]. However, due to the limited performance and complex light-response mechanism of traditional III–V NWs photodetectors, their real potential in practical applications is hindered. The optoelectronic properties of III–V NWs can be modulated by constructing heterostructures and doping.

**Figure 12:**
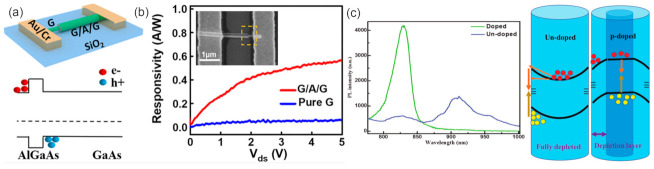
(**a**) Schematic of the G/A/G NW photodetector. (**b**) Energy band diagram of the G/A/G NW channel along the radial direction [136]. Copyright 2020, American Chemical Society. (**c**) Single NW PL spectra of doped and undoped GaAs NWs at 20 K and schematic depicting band-bending effects across the nanowire cross section for (**left**) undoped and (**right**) p-doped GaAs NWs [139]. Copyright 2018, WILEY-VCH Verlag GmbH & Co. KGaA, Weinheim.

Utilizing the intrinsic and valence band offsets of different materials, the 2D electron/hole tube (2DE/HT) structure has been constructed with a GaAs/AlGaAs/GaAs NW (Figure 12a) [136]. The established 2DET at the external AlGaAs/GaAs interface can induce the axial propagation of photogenerated electrons and inhibit radial carrier diffusion. Meanwhile, the photogenerated holes generated by the internal GaAs layer are blocked by the AlGaAs barrier layer and moved to the metal electrode under the action of the electric field, forming 2DHT at the internal AlGaAs/GaAs interface. Th 2DET and 2DHT structures will facilitate efficient carrier transport and collection to achieve the enhanced performance of the GaAs-based photodetector (Figure 12b). Besides, efficient photodetector has also been realized by the core-shell NWs [137,138]. By constructing a type-I heterostructure (GaAs/AlGaAs NW) with a metal-semiconductor-metal radial architecture, the built-in electric fields at the semiconductor heterointerface and at the metal-semiconductor Schottky contact can promote the photogenerated charge separation, leading to enhanced photosensitivity [137].

The surface states of undoped GaAs NWs can act as electron traps and completely deplete the electrons, leading to the upward bending of the conduction band (CB) and valence band (VB). Due to the accumulated photon-generated holes on the NW surface, electrons move to the surface through tunneling, and crossing the barrier will recombine at the surface, leading to a weaker interband emission but longer emission wavelengths. The p-type GaAs NW has shown extraordinary photoelectric properties in photoelectric detection [139]. The surface state of the p-type NW is similar to that of the donor and can trap holes, leading to the negatively charged surface depletion zones and the downward bending of the CB and VB bands (Figure 12c). This effectively restricts photogenic holes to NW centers. Due to the large effective mass and low mobility of photogenic holes, their transport to the NW centers was greatly restricted by this band structure. Therefore, doped NW has greater transport-to-receptor emissions and lower surface-state emissions.

Moreover, due to the distinct refractive index, the nano-photon resonance in WZ and ZB InAs NW arrays showed that the absorption strength of WZ NWs is twice that of ZB NWs, and the resonance of WZ NWs can be reduced to λ ≈ 380 nm when the diameter is reduced, while that of ZB NWs is only λ ≈ 500 nm [114]. With the polarization-resolved and temperature-dependent photoluminescence of ZB and WZ InP NWs, A. Mishra et al. found that the WZ NWs exhibited a bandgap 80meV higher than the ZB NWs. Besides, ZB NWs exhibited strong polarization parallel to the NW axis, while the WZ NWs exhibited polarized emission perpendicular to the NW axis [115].

## 6. Conclusions

Due to the excellent physical properties of III–V materials, lots of attention has been paid to the growth and performance improvement of related electronic devices, while the microscopic understanding of the growth process, structure–property relationship and dynamic structural evolution during application is relatively limited. In this short review, we present a summary of the narrow bandgap III–V NWs from synthesis to application. The role of catalysts/droplets in controlling the morphology and phase during the NW growth process has been dynamically revealed from the atomic level by in situ techniques, which can provide valuable guidance for the controlled synthesis of III–V nanostructures and shed light on the microscopic growth mechanisms. Moreover, some fundamental issues related with the fabrication (contact formation) and application (thermal and chemical environment) of III–V NW-based devices has also been elucidated through in situ techniques. The modulation of structural parameters (orientation, phase, size and defects) on the physical properties like electronic, mechanical and optical properties has been introduced. This information is quite important for the design and performance improvement of III–V NW-based electronics. We also simply introduced the application of III–V materials in the energy storage field. We hope this review can provide some in-depth understanding of the synthesis, design, fabrication and application of III–V nanostructures.
